# Revisiting Metal–Organic Frameworks Porosimetry by Positron Annihilation: Metal Ion States and Positronium Parameters

**DOI:** 10.1021/acs.jpclett.4c00762

**Published:** 2024-04-19

**Authors:** Ahmed G. Attallah, Volodymyr Bon, Kartik Maity, Radosław Zaleski, Eric Hirschmann, Stefan Kaskel, Andreas Wagner

**Affiliations:** † Institute of Radiation Physics, Helmholtz-Zentrum Dresden-Rossendorf, 01328 Dresden, Germany; ‡ Physics Department, Faculty of Science, Minia University, P.O. 61519, Minia, Egypt; § Chair of Inorganic Chemistry I, Technische Universität Dresden, 01062 Dresden, Germany; ∥ Institute of Physics, Maria Curie-Sklodowska University, 20-031 Lublin, Poland

## Abstract

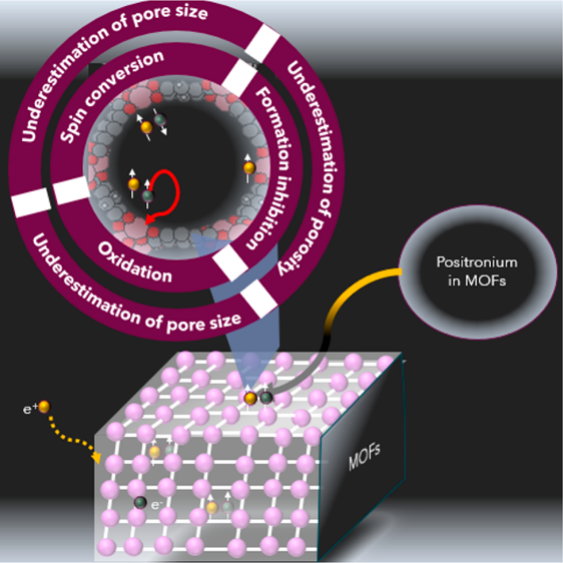

Metal–organic frameworks (MOFs) stand as pivotal porous materials with exceptional surface areas, adaptability, and versatility. Positron Annihilation Lifetime Spectroscopy (PALS) is an indispensable tool for characterizing MOF porosity, especially micro- and mesopores in both open and closed phases. Notably, PALS offers porosity insights independent of probe molecules, which is vital for detailed characterization without structural transformations. This study explores how metal ion states in MOFs affect PALS results. We find significant differences in measured porosity due to paramagnetic or **oxidized** metal ions compared to simulated values. By analyzing CPO-27(M) (M = Mg, Co, Ni), with identical pore dimensions, we observe distinct PALS data alterations based on metal ions. Paramagnetic Co and Ni ions hinder and quench positronium (Ps) formation, resulting in smaller measured pore volumes and sizes. Mg only quenches Ps, leading to underestimated pore sizes without volume distortion. This underscores the metal ions’ pivotal role in PALS outcomes, urging caution in interpreting MOF porosity.

Metal–organic frameworks (MOFs) represent a remarkable class of porous materials characterized by the intricate assembly of metal centers linked to organic linkers through strong bonds, often referred to as reticular synthesis.^1^ MOFs have garnered significant attention in materials science due to their exceptional physical and chemical properties, which include an expansive surface area, low density, substantial pore volume, and active sites that facilitate various guest–host interactions.^2−4^ The versatility of MOFs lies in their ability to adopt numerous structures, all orchestrated through the principles of coordination chemistry.^5^ The wide array of properties and functionalities inherent to MOFs has spurred their adoption in a diverse range of applications. These encompass drug delivery systems,^6,7^ water purification technologies,^8,9^ gas storage solutions,^10,11^ catalysts for chemical interactions,^12,13^ separation processes,^3^ and sensing platforms.^13,14^ Notably, MOFs have seen increasing utilization in the energy sector, particularly in fuel cells, batteries, and supercapacitors, making them a focal point of extensive research.^3^

To harness the full potential of MOFs in these applications, it is essential to employ efficient characterization techniques that are capable of elucidating their chemical and physical attributes comprehensively. For instance, the study of switchable MOFs, which exhibit stepwise alterations in physical and chemical properties, e.g., pore opening, upon external stimuli, has received considerable attention.^15^ But one critical aspect remains relatively uncharted: the quantification of residual porosity in the closed pore phase. Residual porosity may exert a pivotal influence on the dynamics of switchable MOFs. Yet traditional sorption techniques employing probe molecules are not suitable for its investigation as they trigger pore opening, providing distorted insight into the content and significance of residual porosity.

Furthermore, water harvesting with MOFs, notably those based on Zr, has emerged as a vibrant area of research.^16,17^ While techniques such as small and wide angle scattering provide certain information about the pore loading mechanism, they often cannot fully elucidate the precise localization of probe molecules, particularly at elevated water content levels.^18,19^ In addition, scattering techniques are strongly dependent on the scattering power and dynamics of the probe molecules. This limitation hinders a comprehensive understanding of the mechanisms governing water uptake within MOFs.

Moreover, MOFs have attracted much attention as potential adsorbents for CO_2_ capture,^20−22^ which is one of the main greenhouse gases that contribute to global warming and climate change. However, there are still some gaps in our understanding of the CO_2_ adsorption mechanism in MOFs. For instance, how does the presence of water vapor affect both the CO_2_ adsorption capacity and selectivity of MOFs?^23−26^

In this context, positron annihilation lifetime spectroscopy (PALS) has recently emerged as an intriguing and complementary approach within the realm of MOF research.^27−29^ Unlike conventional methods such as X-ray diffraction, neutron diffraction, and physisorption, which are useful in terms of monitoring of the structural changes of the host or evaluation of the accessible porosity for particular probe molecules, PALS can be applied in combination with any probe molecules. It does not necessitate the use of probe molecules to identify pores, relying instead on the diffusion of positrons (the antiparticles of electrons). Compared to small angle scattering techniques, such as SAXS and SANS, often used for evaluation of the porosity mainly in meso- and macropore regime, PALS is independent of scattering lengths, cross sections, and absorption coefficients of atoms and isotopes, which often should be considered in scattering techniques. PALS, therefore, excels at pinpointing open and closed free volumes, especially within the range of micropores and mesopores, spanning 0.3 to 50 nm^30^ (with the highest accuracy lies below 10 nm^31^). Moreover, PALS is a nondestructive technique that can be applied across a spectrum of material forms, including bulk samples, powders, and even liquids through customized sample cells at varying temperatures, pressures, and gas environments. This adaptability in measurement conditions renders PALS particularly suitable for in situ investigations, enabling the exploration of phenomena such as the water adsorption process^18,32^ and the reversible transition (switchability) from closed-to-open pores. Furthermore, conducting in situ PALS measurements during gas adsorption offers a unique perspective to comprehend the complex processes involved, specifically in scenarios related to dry and humid CO_2_ capture. The real-time observation of pore-free volume evolution through PALS during adsorption cycles holds promise for uncovering fundamental information about the mechanisms underlying these processes. Additionally, PALS is well-suited to evaluate porosity in constrained pores, where the pore neck dimensions are smaller than those accessible to probe molecules in traditional adsorption techniques.

However, when working with MOFs containing metal centers, careful consideration is imperative to avoid potential sources of interference, that could lead to misinterpretations in the PALS results. To comprehend these sources and their implications, a more detailed explanation of PALS porosimetry^33^ is warranted.

The foundation of PALS porosimetry rests upon the annihilation lifetime of orthopositronium (o-Ps, see more details in the PALS section in the Supporting Information), a bound state formed by an electron and a positron. o-Ps annihilation can occur intrinsically (with its own electron), but in matter, there is also a high probability of annihilation with electrons from the pore walls (or metals and linkers in MOFs) having an antiparallel spin to that of the positron within the o-Ps state. This *pick-off* annihilation^34^ directly determines the effective o-Ps lifetime in PALS porosimetry. Readers are encouraged to consult the detailed information provided in the PALS “*Principles*” section in the Supporting Information. The rate of pick-off annihilation depends on the size of the pore, with small pores leading to a high rate of annihilation (resulting in a short o-Ps lifetime) due to a higher rate of wall collisions and vice versa. The Tao-Eldrup (TE) model,^35,36^ tailored for micropores, and its extensions (ETE)^34,37^ for micro- and mesopores provide a robust framework to directly correlate the measured o-Ps lifetime and the pore size. This relationship, which focuses solely on o-Ps annihilation through the pick-off process, reveals a consistent and predictable trend in the PALS measurements. However, when o-Ps becomes trapped within a pore containing metal ions, a common occurrence in MOFs, it may undergo quenching through chemical reactions or spin conversion due to the presence of electron (e^–^) acceptors or unpaired electrons.^38^ e^–^ acceptors can break down o-Ps, leading to its lifetime being shorter than expected by ETE, or they can even inhibit o-Ps formation. This effect depends on the concentration of the e^–^ acceptors. In some cases, negligible intensities of o-Ps may be detected. Conversely, spin conversion by unpaired electrons transforms o-Ps into parapositronium (p-Ps) with a very short lifetime. The occurrence of o-Ps quenching by spin conversion undoubtedly has the potential to alter Ps lifetimes (τ) and intensities (*I*), ultimately resulting in an underestimation of pore size (*D*) and volume (*V*_pore_),^39^ respectively. Chemical inhibition, conversely, directly influences the probability of Ps formation, a phenomenon often reflected in the total Ps intensity, *I*_Ps_, leading to a notable reduction in its magnitude.^40^ The reduction in o-Ps lifetimes due to quenching lies in the added o-Ps annihilation channel when quenchers are present. In this case, the measured annihilation rate (λ, inverse the o-Ps lifetime) can be generalized as
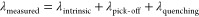
1and it will no longer reflect the pore size only. Applying the ETE model to this lifetime would result in an underestimation of the pore size. This inherent challenge can significantly complicate the reliability of PALS results, especially in applications where PALS is the sole available method or, often, the more informative method of choice. This is of particular significance in applications that involve the investigation of closed pores, during in situ treatments, and during gas or water uptake in MOFs.

So far, no attention has been paid to the influence of the metal nodes in MOFs on the o-Ps lifetime values and intensities, despite metal ions in zeolites,^27^ polymers,^41,42^ and aqueous solutions^43^ having been shown as strong quenchers and inhibitors for o-Ps. In this regard, MOFs with inherent acidity (leading to o-Ps oxidation) and paramagnetic metal nodes require careful consideration of o-Ps quenching and inhibition for accurate interpretation. Therefore, this work is dedicated to illuminating the factors that may disrupt the PALS outcomes in MOF characterization. Urgently addressing these influences is pivotal as it serves to provide immediate guidance for researchers utilizing PALS in MOF studies. Motivated by the discussions presented above, this study presents a systematic investigation into the quenching of o-Ps within the open metal sites of the isostructural MOF members, specifically CPO-27(M) (M = Mg, Co, Ni). (CPO = coordination polymer of Oslo). These frameworks are constructed of corresponding metal ions interconnected by 2,5-dihydroxyterephthalate linkers, which creates a three-dimensional honeycomb-like network with a composition M_2_(dhtp) possessing channel-like pores with 11.5 Å in diameter if coordinated solvent molecules are removed.^44^ With a metal to ligand ratio of 2:1, the CPO-27 series show one of the highest concentrations of the open metal sites among all MOFs, and therefore, they are intensively discussed for the broad range of applications ranging from the hydrogen storage,^45^ carbon dioxide capture,^46^ and gas separation.^47^

*Evidence of o-Ps Quenching in MOFs.* To highlight the influence of o-Ps quenching by metal ions in MOFs, we considered the PALS results found in the literature for MIL-101(Cr),^48^ IFP-8(Co),^49^ HKUST-1(Zn),^50^ and IFP-6(Cd).^51^ Then, we computed the ratio between their pore sizes determined from PALS (*D*_PALS_, see more details in the PALS section in the Supporting Information), assuming pick-off only, and those calculated from crystallographic structure (*D*_XRD_). Figure 1.a illustrates the dependence of the ratio *D*_PALS_/*D*_XRD_ of the above MOF members (red symbols) on the atomic number *Z*. The ratio *D*_PALS_/*D*_XRD_ is a measure of the concordance between PALS porosimetry and crystallographic calculations. In simpler terms, it provides insight into how closely the PALS results align with the crystallographic structure. Obviously, the ratio is less than unity for all selected MOFs and decreases with *Z*. This may indicate that o-Ps quenching (increasing the rate of o-Ps annihilation) occurs and it depends on the metal ion.

**Figure 1:**
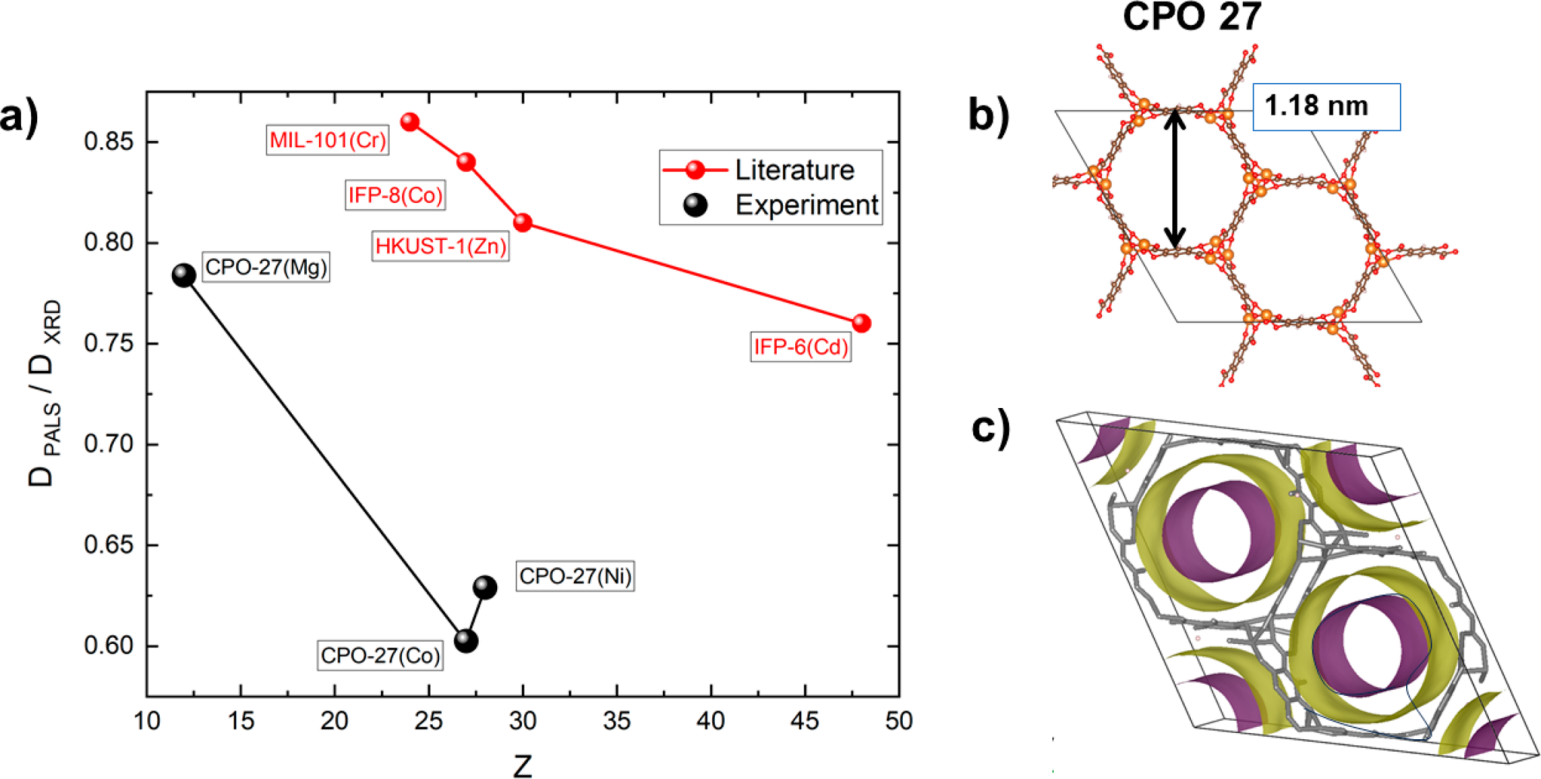
(a) The ratio of pore size calculated from PALS (*D*_PALS_) and from single crystal XRD (*D*_XRD_) as a function of the atomic number of the MOF-metallic nodes. o-Ps lifetimes are taken from the literature (MIL-101(Cr),^48^ IFP-8(Co),^49^ HKUST-1(Zn),^50^ and IFP-6(Cd),^51^ and measured CPO-27(M) (M = Mg, Co, and Ni). (b) Crystal structure from PXRD and the calculated cavity size in CPO-27(M). (c) Visualization of the simulated o-Ps distribution density in CPO-27(M) by assuming no chemical quenching (the purple isodistribution has five times higher Ps density); the corresponding weighted average simulated o-Ps lifetimes are given in Table S.1.

One may think that the difference between *D*_PALS_ and *D*_XRD_ originates not from quenching but from deficiencies of the models,^34−37^ used to correlate the measured o-Ps lifetime to pore size. In these models, the o-Ps exotic atom is considered to be confined and highly localized in a pore with rigid pore walls. In this state, the pick-off annihilation of o-Ps takes place as a result of its interaction with the surrounding pore wall in the closest neighborhood. However, this is not the case in MOFs, where the volume of free space exceeds the volume of material, which is organized more like a colonnade supporting the material structure than like walls. In such an environment o-Ps can be considered as a delocalized Bloch state^52^ that spreads through the large part of the matrix without being confined in a certain pore. Therefore, an adapted model is required to account for the architecture of the MOFs. In that sense, if it is indeed a pure problem of the used models, one would expect longer lifetimes, as the o-Ps atom is nearly free spread over the internal space of MOFs with relatively few pick-off sites, and the probability of pick-off annihilation should be lower than in the case of being confined and localized between pore walls. Even if o-Ps is localized in the relatively shallow potential well of a MOF’s cavity, the surrounding walls are openwork, which would result in the lowering of the electron-density-related Δ parameter (see “*Principles*” in the PALS section in the Supporting Information) of the models and would increase the o-Ps lifetime again.

In order to address the doubts about the necessity to consider the o-Ps quenching in MOFs and to evaluate the validity of the empirical dependence between *D*_PALS_/*D*_XRD_ and *Z* depicted in Figure 1, we compare the experimental results of isostructural CPO-27(M) (M = Mg, Co, and Ni) MOFs with the literature data. Isostructural MOFs share identical topology and pore size but differ only in the type of metal ion, which allows us to isolate the influence of metal ions on o-Ps quenching. To ensure the cleanliness and integrity of the pores against potential moisture intrusion from the surrounding environment during sample transfer and preparation for the PALS experiments, a systematic approach was implemented. Desolvated samples, with emptied pores, were deliberately filled with ethanol. Subsequently, the ethanol-loaded samples were then subjected to controlled heating within the PALS chamber, with the temperature incrementally raised to 180 °C, as thoroughly explained in the PALS “*sample treatment*” section in the Supporting Information. To prove the feasibility of this hypothesis, the porosity of CPO-27(M) frameworks was assessed by nitrogen physisorption on the samples degassed at the conditions used in the PALS studies. Nitrogen physisorption experiments were conducted after each of three desolvation steps at 80, 120, and 200 °C, showing the stepwise successful desolvation of CPO-27 MOFs (Figure S.1, Supporting Information). In the first step at 80 °C, mainly noncoordinated guest molecules could be removed, which is reflected in the good matching with theoretical volume (Figure S.1.d-f, Supporting Information). At 120 °C, a significant portion of coordinated ethanol was removed, confirmed by a pronounced increase in pore volume for all three MOFs. This increase is nicely reflected in the PALS measurement showing a step in τ_3_ and *I*_3_ (o-Ps in framework pores) values between 80 and 120 °C (Figure S.2, Supporting Information). After degassing at 200 °C, the pore volume in all three cases approaches the theoretical values (Figure S.1.d-f, Supporting Information). This means that ethanol is completely removed, and open metal sites are expected to be exposed. This complete pore emptying is also reflected in the PALS results in Figure S.2, where both τ_3_ and *I*_3_ reach high and saturated values in almost all cases. The distinct nonsaturation observed in *I*_3_ for Co^2+^ is noteworthy. Despite its peculiarity, adsorption data suggest that thorough guest removal occurs mainly between 180 and 200 °C.

Only the o-Ps lifetimes in the framework pores (τ_3_, see section “*Equipment and data treatment*” in the Supporting Information for more details) of the ethanol-free samples were used to calculate *D*_PALS_ for CPO-27(M) in Figure 1. Notably, the calculated *D*_PALS_ for the isostructural members of CPO-27(M) vary significantly, testifying that an effect other than pick-off, i.e., quenching, influences the annihilation rate. Moreover, the CPO-27(M) series exhibits a deviation from the behavior observed in samples from the literature, indicating that the o-Ps quenching rate is influenced not only by the atomic number of the metal atoms. These findings highlight the crucial role of the chemical nature of metal ions in the differences illustrated in Figure 1, emphasizing the need to consider interactions between Ps and metal ions within the MOFs. They also indicate that o-Ps quenching in MOFs is a complex phenomenon that depends on various parameters of the metal ion and the linker, e.g., chemistry, access of metal ions from the pore surface, and impurity removal. Thus, the results presented in Figure 1 serve as an indication of the presence of Ps quenching in MOFs, emphasizing the need for caution when interpreting PALS results in the context of MOFs.

*Disentangling o-Ps Quenching.* The next task now is to decouple the origins of o-P quenching, concentrating exclusively on the chemical characteristics within a series of topologically identical CPO-27(M) MOFs. Table S.1 presents the calculated o-Ps lifetimes from simulation within CPO-27(M), based on the crystal structure shown in Figure 1.b, along with the corresponding o-Ps density distribution in Figure 1.c, under the assumption of no quenching. However, quenching induced by chemical reactions or spin conversion will result in correspondingly shorter o-Ps lifetimes and reduced intensities. The impact of metal ion-dependent quenching on o-Ps data becomes evident upon a thorough examination of the individual parameters derived from the PALS data analysis of the emptied pores following complete ethanol removal (described in the “*equipment and data treatment*” section for PALS in the Supporting Information). Table 1 aslo demonstrates the measured o-Ps lifetimes and intensities at RT after in situ ethanol removal from CPO-27(M) (open symbols in Figure S.2). The intensity of the fourth lifetime component (*I*_4_) lies between ∼0.8 and ∼2.1%. The origin of this low-intensity component is peculiar, but it may represent a tiny degree of defective pores due to some deformed linkers in the structure or spaces between crystallites. This component does not appear in the simulation, which considered perfect crystals without such defects or spaces. Since we are aware of the quenching in the framework pores and such defects or spaces can be altered during sample preparation and handling, we focus on the following discussions on τ_3_ and *I*_3_ (the main pore component).

**Table 1:** Measured o-Ps Lifetimes (τ_3_ and τ_4_) and Their Intensities (*I*_3_ and *I*_4_) in CPO-27(M) with Different Metal Ions

	Mg^2+^	Co^2+^	Ni^2+^
τ_3_/ns	3.94 ± 0.01	2.50 ± 0.04	2.65 ± 0.07
*I*_3_/%	37.49 ± 0.13	3.40 ± 0.06	2.60 ± 0.06
τ_4_/ns	37.00 ± 2.20	22.72 ± 1.24	25.65 ± 1.17
*I*_4_/%	2.07 ± 0.05	0.79 ± 0.02	1.47 ± 0.03

The water-free CPO-27(M) series has nonoccupied coordination (open metal) sites,^53−55^ which renders the material highly reactive for guest atoms, o-Ps in this case. Consequently, once situated within the pores, o-Ps will readily discern the presence of metal ions, making them highly susceptible to the chemical characteristics of these metal ions. Given that the resolved o-Ps lifetimes in the main pores (τ_3_ in Table 1) for all samples are consistently shorter than the simulated values (Table S.1), it is reasonable to anticipate o-Ps quenching, either through chemical reactions (such as oxidation, addition, complex formation, etc.) or via spin conversion.^56^ Because the lifetime for each metal ion is different (they follow the order Co^2+^ < Ni^2+^ < Mg^2+^), quenching seems to be dependent on the metal ions. Moreover, the distinct differences between the resolved o-Ps intensities depending on the metal ion suggest that o-Ps formation and annihilation in CPO-27(M) are governed by the chemistry as well. For instance, the o-Ps intensity is significantly diminished in the cases of Co^2+^ and Ni^2+^ compared to Mg^2+^. It is worth highlighting that the pore volumes of all members within the CPO-27(M) series in Figure S.1.d-f, following ethanol removal, are close to each other (∼0.50 cm^3^ g^–1^ for Co^2+^ and Ni^2+^ and ∼0.54 cm^3^ g^–1^ for Mg^2+^), suggesting comparable porosity among them. Therefore, the greatly diminished *I*_3_ in CPO-27(Ni) and CPO-27 (Co) cannot be attributed to lower porosity in these samples; instead, it genuinely underscores the influence of these metal ions on Ps formation and annihilation. To assess the often-underestimated porosity resulting from quenching and spin conversion, we endeavored to derive the pore size distribution (PSD) from PALS results; however, our efforts were in vain. The challenge lies in the fact that determining the pore size distribution requires a comprehensive analysis of PALS data using the expression:^39^
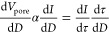
2with  the Ps distribution over lifetimes and  the derivative of τ(D) relation from the ETE model. However, the analysis did not reveal any distribution in all of the CPO-27 measured samples. This absence can be attributed to the cumulative effects of the extremely narrow width of the theoretical PSD (Figure S.3, Supporting Information), ranging between 11.8 and 12.8 Å for fully desolvated samples, and the negligible Ps intensity, especially in the case of Ni^2+^ and Co^2+^.

The presence of paramagnetic ions such as Co^2+^ and Ni^2+^ initiates spin conversion^38,56,57^ because they possess unpaired electrons in their respective d-shells (3d^7^ for Co^2+^, 3d^8^ for Ni^2+^). On the other hand, the Mg^2+^ cation is a closed-shell ion, and thus, no spin conversion of o-Ps is anticipated, as observed in the case of CPO-27(Mg) owing to the absence of unpaired electrons. In spin conversion, the o-Ps with parallel spins of positron and electron are converted to p-Ps with antiparallel spins. The conversion is reflected by the intensities of o-Ps (*I*_3_ + *I*_4_) to p-Ps (*I*_1_) shown in Figure 2.a, where *I*_p-Ps_ > *I*_o-Ps_ for Co^2+^ and Ni^2+^ ions. It should be noted that intensity *I*_1_ represents the intensity of a complex component, which includes at least p-Ps and spin converted o-Ps. It was designated p-Ps to indicate that its dominant origin is the internal annihilation of antiparallel spin Ps (for more details, see the section “*Equipment and data treatment*” in the Supporting Information). This is further evidenced by the ratio of *I*_o-Ps_/*I*_p-Ps_ (Figure 2.b). The lack of spin conversion for Mg^2+^ is indeed confirmed in Figure 2.a, as evidenced by the notable *I*_3_ (o-Ps intensity) at 41%, which surpasses that of p-Ps and is larger than those in most porous materials. Moreover, the metal ions in the CPO-27(M) series are known to act as Lewis acid centers.^58,59^ Lewis acids are known to inhibit Ps formation by scavenging either electrons or positrons.^27^ To verify this, we perform a calculation of the total Ps intensity (*I*_Ps_ = *I*_p-Ps_ + *I*_o-Ps_ = *I*_1_ + *I*_3_ + *I*_4_) and present the results in Figure 2.a against the respective metal ions. The data strongly suggest that Co^2+^ and Ni^2+^ ions trigger Ps inhibition, as evidenced by their remarkably lower total Ps intensity compared to Mg^2+^. This reduction in total Ps intensity implies the involvement of Co^2+^ and Ni^2+^ ions in inhibiting Ps formation, an effect not attributed to spin conversion alone, which should keep *I*_Ps_ at a similar level in all CPO-27(M). Conversely, even though Mg^2+^ is also known for its Lewis acidity, Figure 2.a illustrates a total Ps intensity reaching approximately 75%. This difference in *I*_Ps_ indicates a distinct behavior compared to that of Co^2+^ and Ni^2+^ ions concerning Ps inhibition. This observation suggests that the hindrance or absence of Ps inhibition occurs when Mg^2+^ and possibly other diamagnetic ions are present in the MOFs.

**Figure 2:**
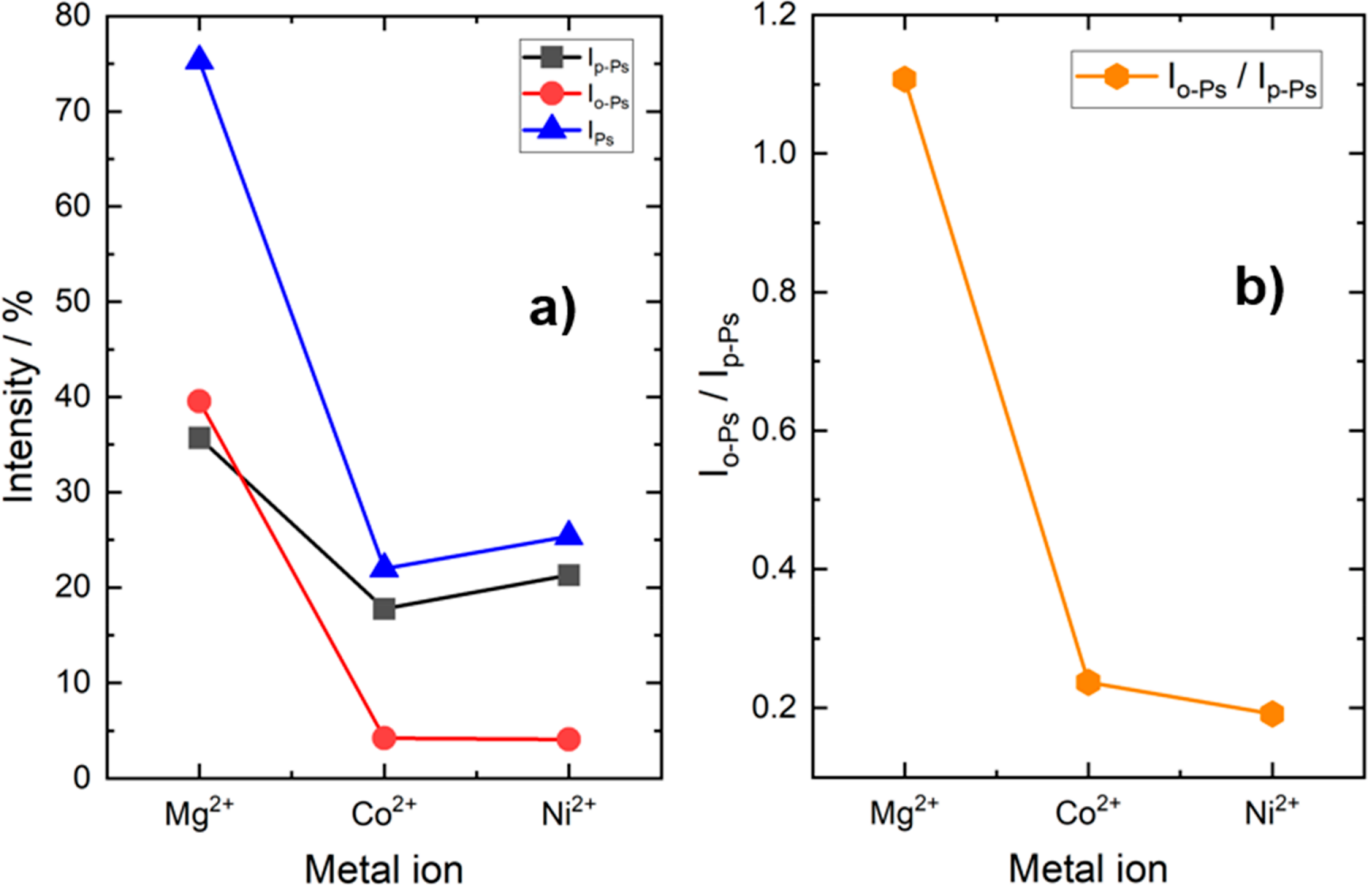
(a) Intensities of p-Ps, o-Ps, and total Ps (*I*_Ps_ = *I*_p-Ps_ + *I*_o-Ps_) and (b) ratio between *I*_o-Ps_ and *I*_p-Ps_ as functions of the metal ion in CPO-27 MOFs. Notably, the resolved *I*_1_ was employed to emphasize *I*_p-Ps_ in the figure, given that p-Ps constitutes the primary contributor to *I*_1_ (as discussed in the text and in the Supporting Information), while *I*_3_ + *I*_4_ represents the intensity of o-Ps.

If more than one type of o-Ps quenching exists, the total quenching annihilation rate is the sum of the individual rates.^60^ This means that λ_measured_ in eq 1 will be larger (and the o-Ps lifetime will be shorter) with multiple quenching sources. This explains the τ_3_ values in Table 1. Presumably, the quenching effect of Mg^2+^ (resulting in a measured lifetime of 3.94 ns against the simulated 6.27 ns, Table S.1) is attributed solely to a chemical reaction involving o-Ps oxidation by this ion, according to the reaction
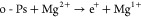
3

On the other hand, the cumulative quenching effect, stemming from oxidation, spin conversion, and inhibition, in Co^2+^ and Ni^2+^ can be the cause of the shorter-than-expected lifetimes and diminished intensities presented in Table 1. Therefore, one can generalize the quenching annihilation rate from eq 1 in CPO-27(M) as
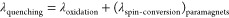
4

The expected sources of quenching based on the type of metal ion in CPO-27(M) are summarized in Figure 3. This visualization portrays the consequences of acidity in Mg^2+^ (leading to a reduction in the o-Ps lifetime) and the combined effects of acidity, spin conversion, and inhibition in Co^2+^ and Ni^2+^ (resulting in reductions in both lifetime and intensity).

**Figure 3:**
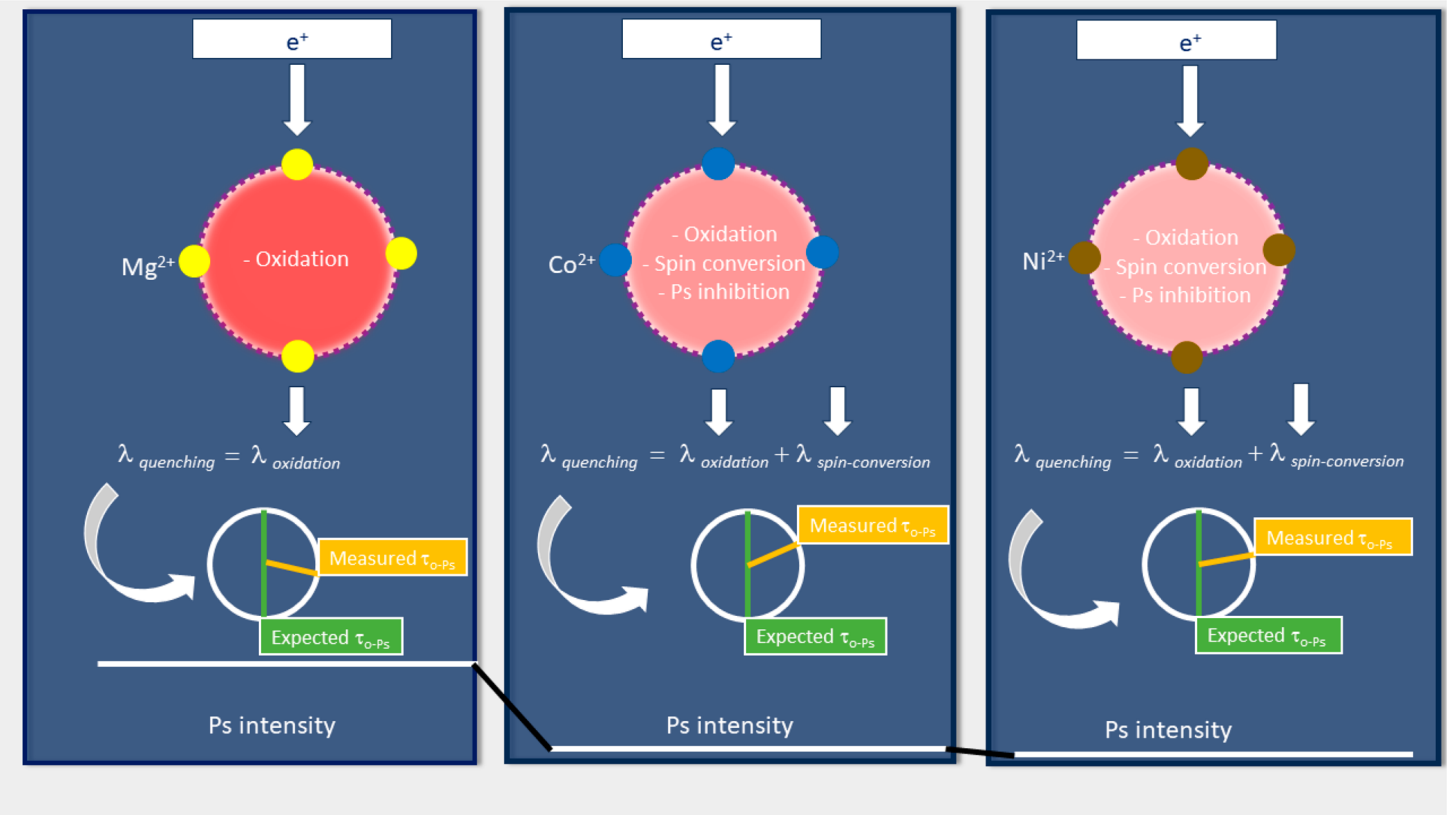
Visualization of the expected impacts of various metal ions within MOFs on Ps characteristics, encompassing the straightforward quenching attributed to oxidation in the presence of acidic centers (Mg^2+^), and the collective impact of acidity, spin conversion, and Ps inhibition (the negligible Ps intensities represented by the horizontal lines in the bottom of the figure) in the context of paramagnetic ions (Co^2+^ and Ni^2+^). The brightness of the red color reflects the Ps intensity inside the pores.

In conclusion, this exploration elucidates the substantial impact of metal ions within MOFs on the PALS results. The study, focusing on the CPO-27(M) series (M = Mg, Co, Ni), emphasizes the intricate relationship between the chemical nature of these ions and the apparent porosity of MOFs as assessed by PALS. This influence becomes apparent through a comparison of simulated o-Ps lifetime magnitudes and pore volumes from gas adsorption alongside the measured values. Additionally, this research strengthens our understanding of o-P interactions in MOFs by systematically identifying and dissecting quenching mechanisms including oxidation, spin conversion, and inhibition. These mechanisms exhibit distinct and pronounced effects based on the specific metal ions. For example, paramagnetic ions like Co^2+^ and Ni^2+^ trigger both spin conversion and oxidation, resulting in shorter o-Ps lifetimes, while diamagnetic ions like Mg^2+^ lead to quenching solely via Ps oxidation, creating unique PALS signatures.

These observations underscore the complexity of o-Ps quenching and emphasize the necessity for a comprehensive understanding of metal ion chemistry to precisely interpret PALS data in MOF characterization. They also prompt careful consideration and caution in assessing the chemical properties of metal ions, providing crucial insights for refining the evaluation of porosity using PALS within MOFs and enhancing their characterization across diverse applications. Looking ahead, the prospect of implementing metal-selective shielding to alleviate o-Ps quenching in MOFs with open metal sites emerges as a promising avenue for future exploration. This pragmatic approach, which is devoid of substantial alterations to porosity or undue complications in sample preparation, holds considerable potential.

## Supporting Information Available

Information on experimental details including sample preparation, gas adsorption, powder X-ray diffraction, SEM, and comprehensive details about the PALS method, equipment, data acquisition and analysis, PALS result of in situ methanol removal from the CPO-27(M) series, and Monte Carlo simulation to calculate the o-Ps lifetimes in the structures (PDF)
