# Supplementary material for: Revisiting Metal–Organic Frameworks Porosimetry by Positron Annihilation: Metal Ion States and Positronium Parameters
Source: J Phys Chem Lett. 2024 Apr 19;15(17):4560–7. doi: 10.1021/acs.jpclett.4c00762 (PMC11071070; doi:10.1021/acs.jpclett.4c00762)
Supplement: Supplementary file 1 — jz4c00762_si_001.pdf [file jz4c00762_si_001.pdf]

Supporting Information

Revisiting Metal-Organic Frameworks Porosimetry by Positron Annihilation: Metal Ion States and Positronium Parameters

Ahmed G. Attallah[1, 2,], Volodymyr Bon[3], Kartik Maity[3], Radosław Zaleski[4], Eric Hirschmann[1], Stefan Kaskel[3], Andreas Wagner[1]*

[1]Institute of Radiation Physics, Helmholtz-Zentrum Dresden-Rossendorf, Dresden, Germany

[2]Physics Department, Faculty of Science, Minia University, Egypt

[3]Inorganic Chemistry I, Technische Universität Dresden, Germany

[4]Institute of Physics, Maria Curie-Sklodowska University, Lublin, Poland

Corresponding authors' email addresses: a.elsherif@hydr.de (Ahmed G. Attallah)

Revisiting Metal-Organic Frameworks Porosimetry by Positron Annihilation: Metal Ion States and Positronium Parameters

Ahmed G. Attallah[1, 2,*], Volodymyr Bon[3], Kartik Maity[3], Radosław Zaleski[4], Eric Hirschmann[1], Stefan Kaskel[3], Andreas Wagner[1]

[1]*Institute of Radiation Physics, Helmholtz-Zentrum Dresden-Rossendorf, Dresden, Germany*
[2]*Physics Department, Faculty of Science, Minia University, Egypt*
[3]*Inorganic Chemistry I, Technische Universität Dresden, Germany*
[4]*Institute of Physics, Maria Curie-Sklodowska University, Lublin, Poland*

Experimental details

Materials

$Mg(NO_3)_2 \cdot 6H2O$, $Co(NO_3)_2 \cdot 6H2O$, $Ni(NO_3)_2 \cdot 6H2O$, and 2,5-dihydroxyterephthalic acid were purchased from Sigma Aldrich and used in the synthesis without pre-treatment. CPO-27(Mg), CPO-27(Co), and CPO-27(Ni) were synthesized following previously published procedures.[1–3] The phase purity, porosity, crystal size and morphology were confirmed in Fig. S.1.

Structural and textural characterization

Nitrogen physisorption experiments. Physisorption of nitrogen (99.999 %), at 77 K was performed on a BELSORP-max instrument of MICROTRAC MRB. As equilibrium conditions a pressure change of 1% upon 300 s was chosen for each point of the isotherm. The dead volume was determined using helium (99.999 %). The values for adsorbed gases are given at standard conditions (273.15 K, 101.325 kPa) in mmol/g. The Dewar with liquid nitrogen was used to keep the sample's temperature at 77 K. The standard 6 mm adsorption cells were used in all experiments. In order to reproduce the desolvation conditions, used for PALS measurements as described below, pre-activated samples of CPO-27(Mg), CPO-27(Co) and CPO-27(Ni) were first degassed in a vacuum at 280°C in order to completely remove the coordinated water/solvent molecules from the metal centers. After that, the samples were loaded with ethanol at room temperature from the gas phase until the adsorption equilibrium was reached p ~ 7 kPa. In the next step, the nitrogen gas was added until reaching 100 kPa. Then the samples were degassed at 80°C, 120°C, and 200°C in a dynamic vacuum (2 hours/step) and subjected to nitrogen physisorption at 77K (Fig.S.1.a-c). We

selected these temperatures because PALS results on in situ removal of ethanol in Fig.S.2 indicate a transition in o-Ps lifetimes and intensities of all samples between 80 and 120°C and 200°C is close to the end temperature of PALS measurements.

Calculations of geometrical porosity. Geometrical pore volume for CPO-27(M) structures (M – Mg, Co, Ni) was calculated using Zeo++ software.[4] The crystal structures of desolvated frameworks were downloaded from the CCDC database. The structures with ethanol, coordinated to open metal sites were simulated in Materials Studio 5.0 and optimized using geometry optimization procedure and UFF force field. The calculated values are provided in Fig.S.1.d-f, indicating the reduction of the pore volume roughly by a factor of two in the structures with coordinated ethanol.

SEM. The particle size distribution and morphology of the synthesized materials were analyzed using a Hitachi S-4800 and SU-8000 scanning electron microscope (SEM). SEM images are presented in Fig.S.1.g-i

Powder X-ray diffraction. The samples for powder X-ray diffraction (PXRD) analysis were placed as a flatbed sample between two MPET foils (space blanket), connected by vacuum grease. Theoretical PXRD patterns were calculated based on the crystal structures, published in CCDC, using Mercury 3.9 software package. PXRD patterns were collected in transmission geometry on STOE STADI P diffractometer, equipped with line focus Cu X-ray tube, operated at 40 kV / 30 mA, and focusing Ge (111) monochromator (λ = 0.154059 nm) and MYTHEN2 100K (DECTRIS) detector. XRD patterns of the ethanol-desolvated samples, along with theoretical calculation, are depicted in Fig.S.1.j

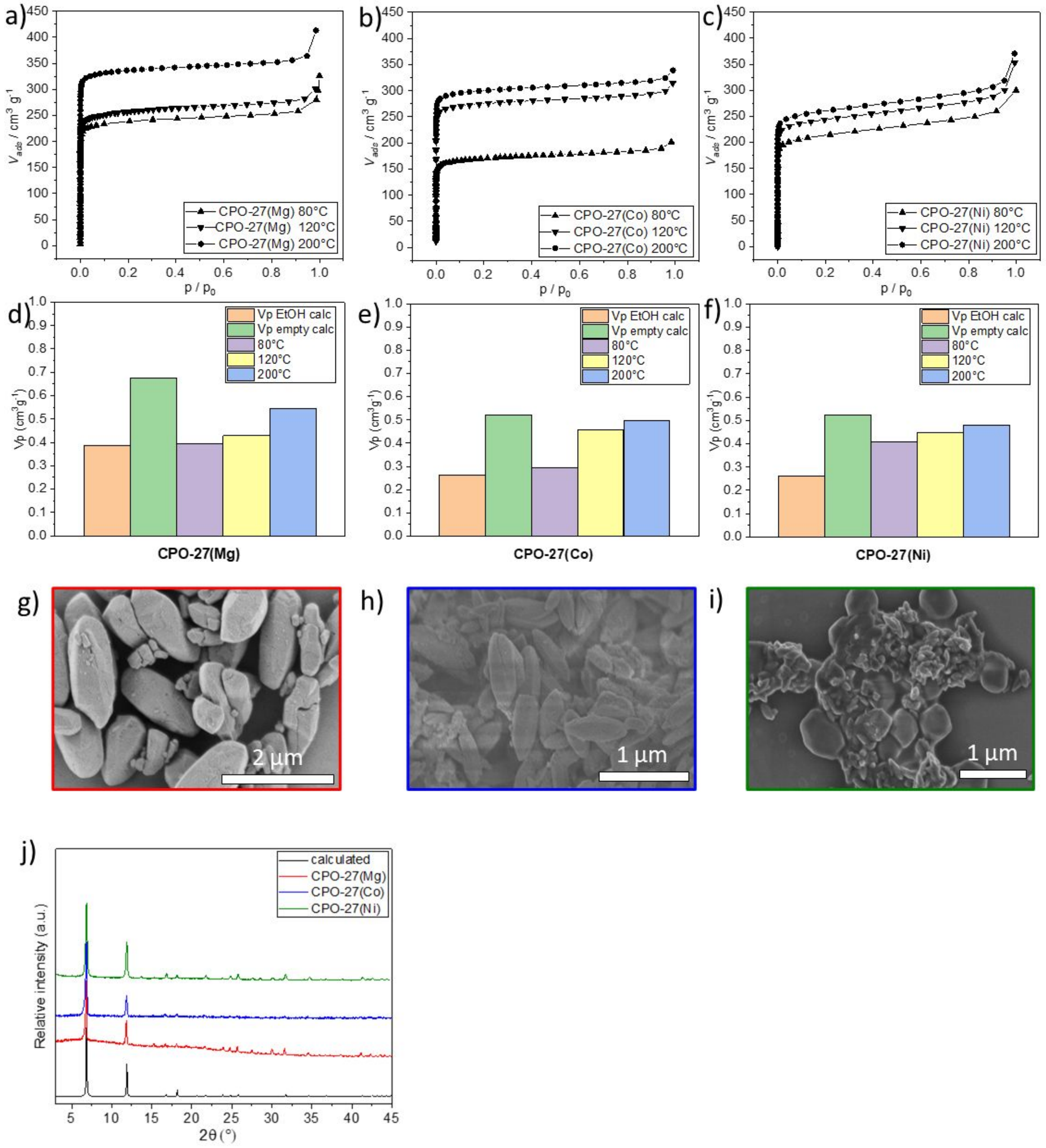

Figure S.1. Standard characterization of CPO-27(M) frameworks: a-c) Nitrogen physisorption at 77K on CPO-27(M) samples desolvated from ethanol at 80°C, 120°C and 200°C; d-f) Theoretically calculated and experimentally derived pore volumes for CPO-27(M) samples; g-f) SEM images for CPO-27(Mg), CPO-27(Co) and CPO-27(Ni) samples; j) Theoretically calculated and experimentally measured XRD on the completely desolvated samples.

Free volume evaluation by positron annihilation lifetime spectroscopy (PALS)

Principles. In PALS, an energetic positron (e^+) interacts with the atoms of matter, ionizing and exciting molecules, until it reaches thermal energy, then it diffuses seeking open volume sites. According to the spur model,[5] thermalized e^+ can bind to one of the ionized electrons (e^-) forming the so-called positronium (Ps) atom in materials containing free volumes e.g. MOFs. The Ps atom exists in either of two states depending on the relative spin alignment of e^- and e^+; a spin antiparallel singlet state or *para*-Ps (*p*-Ps) and a spin parallel triplet state or *ortho*-Ps (*o*-Ps). *p*-Ps self-annihilates with a characteristic lifetime of 0.125 ns and is barely influenced by the medium or the pore size, hence, it cannot be used to evaluate the porosity. On the other hand, *o*-Ps has an intrinsic vacuum lifetime of 142 ns, while in pores e^+ of *o*-Ps and e^- of the pore wall with antiparallel spin can annihilate with a lifetime significantly shorter than 142 ns. This process is known as *pick-off* annihilation.[6] The probability of *pick-off* annihilation depends on the pore size, the larger the pores, the lower the annihilation probability and vice versa. This property is used to correlate the *pick-off* annihilation lifetime of *o*-Ps to pore size.[6–9]

The Tao-Eldrup (TE) model is employed to convert the fitted o-Ps pick-off lifetimes into micropore radii ($R_{3,4,\ldots}$).[8,10] The Tao-Eldrup model replaces a spherical pore, initially characterized by a rectangular potential well with a radius R and a finite potential barrier, with a well featuring an infinite potential barrier and a dimension of R + Δ. This modification enables the wavefunction Ψ(r) of the o-Ps in the ground state of the infinite potential well to interact with the bulk material (electron layer within the pore wall) within the span from R to R + Δ. Δ is employed as an empirical parameter in this model. Then, the o-Ps pick-off annihilation rate equals the annihilation rate in the bulk multiplied by the probability of finding the Ps outside the potential well. The integration of $|\Psi(r)|^2 * r^2$ from R (where the electron density begins to be non-zero, marking the boundary of the finite potential well) to R + Δ computes this probability. This approach enabled the correlation of o-Ps lifetimes within micropores of radius R as

$$\lambda_{TE} = \lambda_a\left[1 - \frac{R}{R+\Delta} + \frac{1}{2\pi}\sin\left(\frac{2\pi R}{R+\Delta}\right)\right] \qquad (S.1)$$

where $\lambda_a = 2$ ns^{-1} is the spin average annihilation rate in the bulk as one-quarter of Ps annihilates as p-Ps of 0.125 ns lifetime and three-quarters annihilates as o-Ps of 142 ns.

In such small pores (narrow potential wells), the spacing between the energy levels is extremely large and o-Ps cannot occupy high energy levels. However, in larger pores, the presence of excited states has to be taken into account. Consequently, the TE model is adapted for larger pore sizes and different pore geometries through the extended TE models (ETE).[11,12] Given that the fitted lifetime values in the present study are anticipated to correspond to micropores (τ_3) and mesopores (τ_4) (further elaborated below), we employed the ETE model.

The ETE model describes the behavior of the Ps in larger pores by solving higher orders of the Bessel function. The probability of the Ps accommodating any energy state is given by Boltzmann statistics. According to the ETE model, the total annihilation rate, λ, of o-Ps is given by

$$\lambda_{\mathrm{ETE}} = \lambda_{2\gamma} + \lambda_{3\gamma} \qquad \text{(S.2)}$$

where $\lambda_{2\gamma}$ is the pick-off annihilation rate in micro- and mesopores, and $\lambda_{3\gamma}$ is the intrinsic o-Ps lifetime in a vacuum (142 ns, because in quite big pores, o-Ps can behave as if it exists in a vacuum and may no longer interact with electrons in pore walls). According to Zaleski and co-workers,[13] the observed annihilation rate constant within spherical pores in the ETE model can be represented as:

$$\lambda_{\mathrm{ETE}} = \frac{\sum_i \lambda_i \, g_i \, e^{\frac{-E_i}{KT}}}{\sum_i g_i \, e^{\frac{-E_i}{KT}}}, \qquad \text{(S.3)}$$

where λ_i, g_i, and E_i are decay constant, statistical weight ($g_i = 2l+1$ for spherical geometry and $g_0 = 1$, $g_m \neq 0 = 2$ for cylindrical geometry), and energy for i-th level, respectively. Following the discussion of the TE model to calculate the total annihilation probability by integrating the wavefunction over the boundary conditions, one gets for spherical pores (as the case in the presented work)

$$\lambda_{\mathrm{ETE}} = \lambda_a \frac{\int_{\chi_{nl}\frac{R}{R+\Delta}}^{\chi_{nl}} j_i^2 \, r^2 \, dr}{\int_0^{\chi_{nl}} j_i^2 \, r^2 \, dr}, \qquad \text{(S.4)}$$

where χ_{nm} is the ith nodes of the Bessel functions j_i, R is the pore size of a spherical pore, and $\lambda_a = 2\ \mathrm{ns}^{-1}$ as defined in eq. S.1. The empirical parameter Δ is slightly dependent on the electron density

of pore walls. In the current work, we used the EELViS code,[14] which solves equation S.4, incorporating micro- and mesopores.

Equipment and data treatment. PALS measurements were conducted using a digital positron lifetime spectrometer equipped with a digitizer (Acqiris DC 282, 10-bit vertical resolution) and three photomultiplier tubes (PMTs, SCIONIX HOLLAND) having three Ce_2Br_3 scintillators. Two scintillators had sizes of Ø=51 mm and h=10 mm, while the third scintillator had Ø=51 mm and h=25.4 mm. The scintillators were positioned to ensure effective coverage of the spatial area around the sample holder and to lower the detection of any backscattered photons by the high-density scintillators. Two of the PMTs were positioned horizontally at a 90° angle from each other, with their front faces separated by roughly 20 mm. The third PMT was vertically aligned, approaching the sample holder from above. Customized data acquisition software [15] facilitated the simultaneous detection of the start and stop signals for each of the three PMTs. This unique feature allowed us to collect six spectra in a single measurement session, significantly, together with the high detection efficiency of Ce_2Br_3 scintillators, reducing the measurement time compared to traditional 2-tube spectrometers. The sample holder was specifically designed to accommodate around 0.4 ml of powdered sample, which surrounded a 20 µCi ^{22}Na positron source sealed within a 5 µm Kapton foil (DuPont). The time range was set to 500 ns in the coincidence box and 600 ns in the PALS data acquisition software, with a channel width of 5 ps. The PALS spectra were analyzed by fitting exponential decay curves to the histograms of recorded time differences, revealing lifetime components (τ_n) with their relative fractions or intensities (I_n). The analysis revealed that a reliable fit, characterized by minimal residual and a χ^2 value of 1.03 – 1.08, was achieved solely when considering four lifetime components. The shortest-lived component, τ_1 of I_1 can be considered as a complex component. Typically, the short-lived component may contain contributions from: free positrons annihilating in atomic defect-free bulk or monovacancies, para-positronium, and spin-converted ortho-positronium. However, due to the physical nature of MOF structures (metal nodes connected by long organic linkers), free positron annihilation in monovacancies or bulk is highly unlikely, with the short-lived component predominantly indicative of p-Ps annihilation (namely Ps intrinsic annihilation). The resolved lifetime components were categorized according to the magnitudes of their lifetimes as: corresponded to *p*-Ps annihilation ($\tau_1 \sim 0.12 – 0.18$ ns), originated from unbound e^+ annihilation ($\tau_2 \sim 0.35 – 0.50$ ns), and the longest-lived components signified the *o*-Ps annihilation within the pores ($\tau_3 > 1$ ns). τ_4 is believed to reflect o-Ps annihilation between

crystals or in deformed pores. In the subsequent discussions, our focus primarily rested on the pore-related lifetime, τ_3 in the framework. This analysis was performed using the PALSFit routine [16], which deconvolutes the spectra from the resolution functions, background, and source contribution. Al and Sn reference samples with known lifetimes were used to estimate the resolution functions and source contribution. About 10.60 % source contribution (shared between Kapton foil (0.382 ns / 10.40 %) and glue (2.60 ns / 0.20 %)) and an average time resolution of 0.28 ns (full width at half maximum) have been determined during the fitting process.

Sample treatment. Desolvated CPO-27 materials exhibit open metal sites that are sensitive to moisture and readily adsorb water due to interactions with the coordinatively unsaturated metal nodes. This poses a challenge for our study, which aims to investigate the impact of unshielded metal nodes on PALS characteristics. To address this issue, an in-situ desolvation process proves to be the most suitable choice, as it ensures the preservation of clean pores during PALS measurements. However, conducting direct desolvation within the PALS chamber is not feasible due to the specific heating program required for CPO-27 desolvation. This program involves a gradual temperature increase, starting with 150 °C overnight, followed by 200 °C for 2 hours, and then 270 °C for 1 hour. Regrettably, this heating program is incompatible with the ^{22}Na positron source, which is sealed in a Kapton foil using epoxy glue and could begin to degrade at temperatures approaching 200 °C. In order to maintain the inner porosity of CPO-27 intact and prevent any source damage, we adopted a two-step approach. First, we performed ex-situ desolvation of the samples according to the required protocol. Subsequently, we loaded the samples with ethanol, which effectively occupied all pores, preventing any foreign substances from entering them during the sample transfer and preparation for PALS measurements. The ethanol-loaded samples were then subjected to in-situ heating inside the PALS chambers, reaching a temperature of ~180 °C. This step allowed for the evaporation of ethanol at reachable temperature with the PALS chamber and the restoration of open-metal sites for our analysis. This in situ ethanol evaporation process was conducted under a dynamic vacuum better than 10^{-6} kPa from RT to 180 °C with 2 hours/step. At each heating step, PALS spectra were obtained with at least 2×10^6 events. The PALS results depicting the ethanol evaporation process are presented in Fig. S.2. These findings convincingly affirm that a temperature up to 180 °C is sufficient to completely purge the pores, as manifested by the consistent stability of o-Ps parameters observed. It also aligns with the findings of the adsorption data in Fig.S.1.

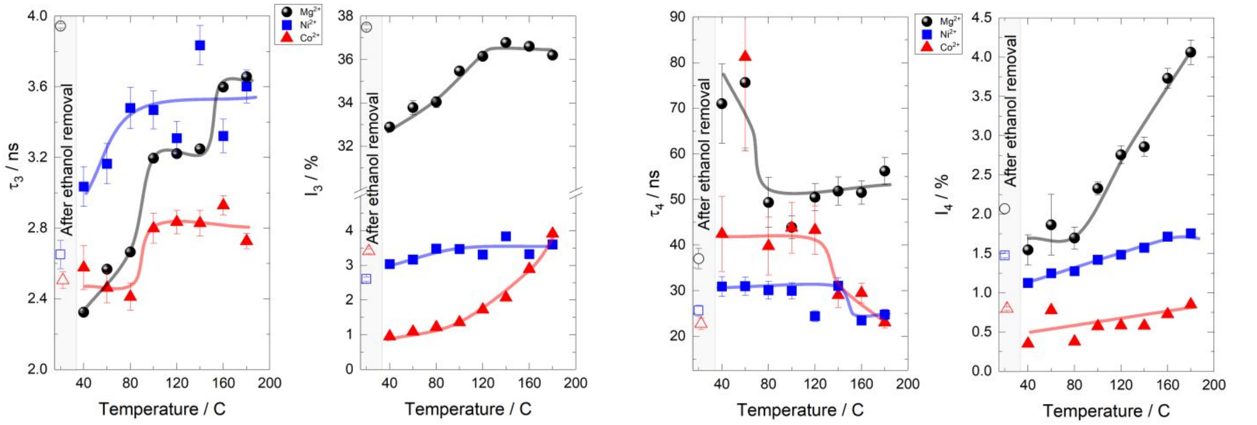

Figure S.2. Evolution of o-Ps lifetimes (τ_3 and τ_4) and intensities (I_3 and I_3) during in-situ ethanol removal from CPO-27 MOFs featuring Mg^{2+}, Co^{2+}, and Ni^{2+} metal ions. Open symbols indicate RT o-Ps lifetimes and intensities of the samples after ethanol removal. The lines are only eye guides.

Theoretical pore size distribution calculated by Zeo++ software[17]

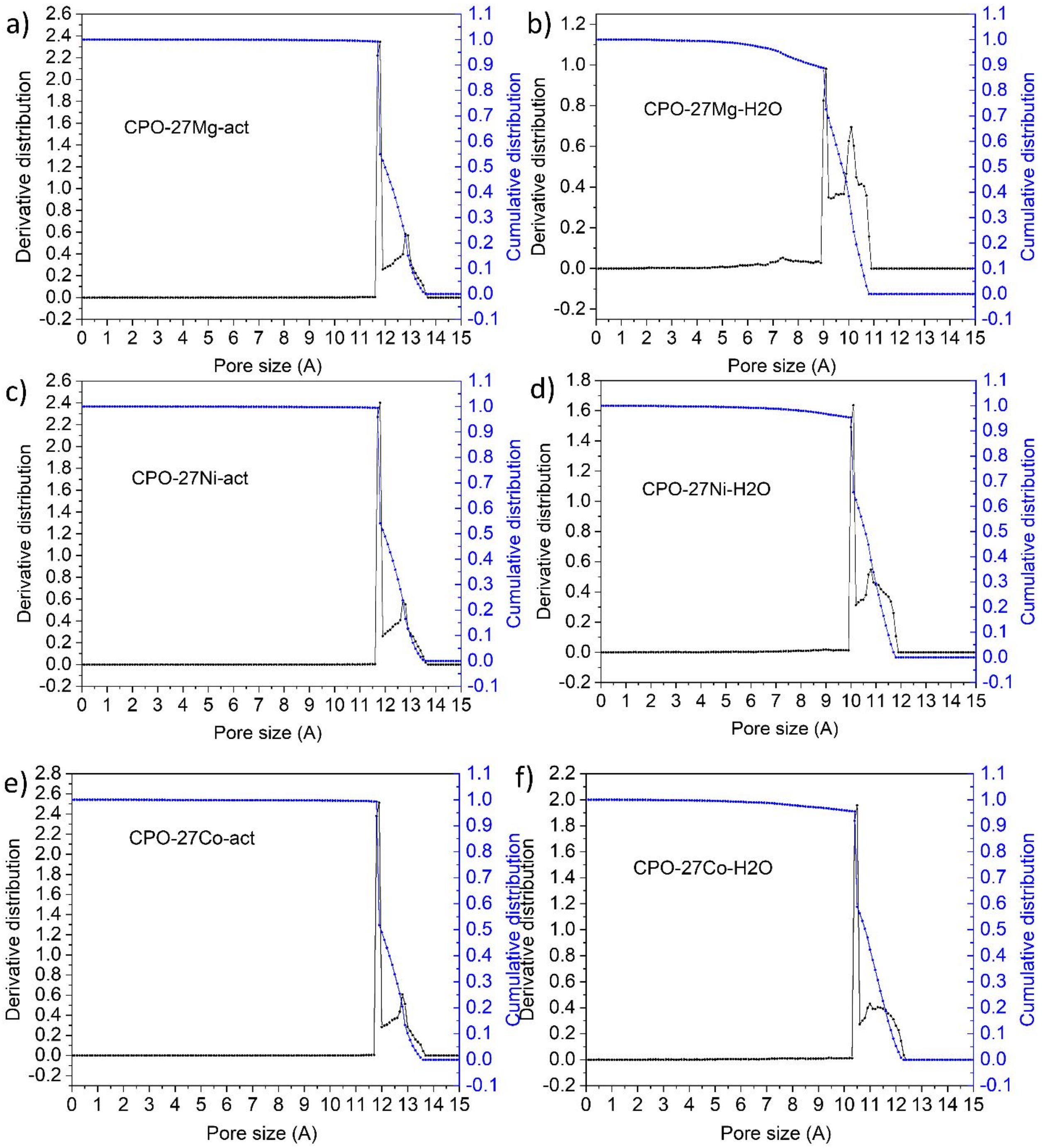

Figure S3. Theoretically calculated pore size distribution for CPO-27 solids: a) CPO-27-Mg-activated; b) CPO-27-Mg-H_2O; c) CPO-27-Ni-activated; d) CPO-27-Ni-H_2O; e) CPO-27-Co-activated; f) CPO-27-Co-H_2O.

Simulating the o-Ps within the CPO-27(M)

In order to calculate the Ps density distribution within the CPO-27(M) series, the simulation uses as input the atom structure of the MOF from PXRD. The total potential felt by the Ps atom is calculated by assuming that a Tao potential is induced by each atom.[18] The Tao potential is zero except for a forbidden region around each atom where it is infinite . It prevents the Ps from entering the inner core of the atom. The size of the forbidden region is proportional to the atom ionic radius. The Tao potential is a good approximation for open structures like MOFs. Additionally, Ps distributes along the whole structure and cannot localize in small and large cavities separately. For a given total potential, the Schrödinger equation for a single Ps is solved numerically.

The forbidden region around each atom is an atomic layer of 0.13 nm thickness, which is the value that produced the best fit in a previous work.[18] The pick-off annihilation rate of Ps is obtained from the overlap integral of the Ps and the electrons in the atomic layer. The lifetime values represent a "weighted average" of the annihilation inside each type of cavity. Oxidation of the Ps atom by positively charged ions within the framework of "chemical quenching" and spin conversion are not included in the calculated annihilated rates, neither atom bonding effects nor delocalized electrons. By running the simulation, we got the Ps density described in Fig.1.c and the simulated lifetimes are given in Table S.1. Table S.1 also contains the measured o-Ps lifetimes and estimated quenching rates for comparison.

Table S.1. The calculated o-Ps lifetimes from simulations, without considering any chemical quenching, measured o-Ps lifetimes, and calculated quenching rates in CPO-27(M) series with different metal ions.

Metal ion	Simulated o-Ps lifetimes (ns)	Measured o-Ps lifetimes (ns)	Quenching rate $\lambda_{measured}$ (1/ns)
Mg^{2+}	6.27	3.94 ± 0.01	0.413 ± 0.001
Co^{2+}	6.63	2.50 ± 0.04	0.551 ± 0.009
Ni^{2+}	6.61	2.65 ± 0.07	0.529 ± 0.014

References

(1) Dietzel, P. D. C.; Blom, R.; Fjellvåg, H. Base-Induced Formation of Two Magnesium Metal-Organic Framework Compounds with a Bifunctional Tetratopic Ligand. *Eur. J. Inorg. Chem.* **2008**, *2008* (23), 3624–3632. https://doi.org/10.1002/EJIC.200701284.

(2) Dietzel, P. D. C.; Morita, Y.; Blom, R.; Fjellvåg, H.; Dietzel, P. D. C.; Blom, R.; Morita, Y.; Fjellvåg, H. An In Situ High-Temperature Single-Crystal Investigation of a Dehydrated Metal–Organic Framework Compound and Field-Induced Magnetization of One-Dimensional Metal–Oxygen Chains. *Angew. Chemie Int. Ed.* **2005**, *44* (39), 6354–6358. https://doi.org/10.1002/ANIE.200501508.

(3) Dietzel, P. D. C.; Panella, B.; Hirscher, M.; Blom, R.; Fjellvåg, H. Hydrogen Adsorption in a Nickel Based Coordination Polymer with Open Metal Sites in the Cylindrical Cavities of the Desolvated Framework. *Chem. Commun.* **2006**, No. 9, 959–961. https://doi.org/10.1039/B515434K.

(4) Willems, T. F.; Rycroft, C. H.; Kazi, M.; Meza, J. C.; Haranczyk, M. Algorithms and Tools for High-Throughput Geometry-Based Analysis of Crystalline Porous Materials. *Microporous Mesoporous Mater.* **2012**, *149* (1), 134–141. https://doi.org/10.1016/J.MICROMESO.2011.08.020.

(5) Mogensen, O. E. Spur Reaction Model of Positronium Formation. *J. Chem. Phys.* **1974**, *60* (3), 998–1004. https://doi.org/10.1063/1.1681180.

(6) Dull, T. L.; Frieze, W. E.; Gidley, D. W.; Sun, J. N.; Yee, A. F. Determination of Pore Size in Mesoporous Thin Films from the Annihilation Lifetime of Positronium. *J. Phys. Chem. B* **2001**, *105* (20), 4657–4662. https://doi.org/10.1021/jp004182v.

(7) Tao, S. J. Positronium Annihilation in Molecular Substances. *J. Chem. Phys.* **1972**, *56* (11), 5499–5510. https://doi.org/10.1063/1.1677067.

(8) Eldrup, M.; Lightbody, D.; Sherwood, J. N. The Temperature Dependence of Positron Lifetimes in Solid Pivalic Acid. *Chem. Phys.* **1981**, *63* (1–2), 51–58. https://doi.org/10.1016/0301-0104(81)80307-2.

(9) Goworek, T.; Ciesielski, K.; Jasińska, B.; Wawryszczuk, J. Positronium in Large Voids. Silicagel. *Chem. Phys. Lett.* **1997**, *272* (1–2), 91–95. https://doi.org/10.1016/S0009-2614(97)00504-6.

(10) Tao, S. J. Positronium Annihilation in Molecular Substances. *J. Chem. Phys.* **1972**, *56* (11), 5499–5510. https://doi.org/10.1063/1.1677067.

(11) Dull, T. L.; Frieze, W. E.; Gidley, D. W.; Sun, J. N.; Yee, A. F. Determination of Pore Size in Mesoporous Thin Films from the Annihilation Lifetime of Positronium. *J. Phys. Chem. B* **2001**, *105* (20), 4657. https://doi.org/10.1021/jp004182v.

(12) Goworek, T.; Ciesielski, K.; Jasińska, B.; Wawryszczuk, J. Positronium States in the Pores of Silica Gel. *Chem. Phys.* **1998**, *230* (2–3), 305–315. https://doi.org/10.1016/S0301-0104(98)00068-8.

(13) Sniegocka, M. ´; Jasi´nskajasi´nska, B.; Wawryszczuk, J.; Zaleski, R.; Deryy Lo-

Marczewska, A.; Skrzypek, I. Testing the Extended Tao-Eldrup Model. Silica Gels Produced with Polymer Template. **2005**, *107*.

(14) Zaleski, R.; Wawryszczuk, J.; Goworek, T. Pick-off Models in the Studies of Mesoporous Silica MCM-41. Comparison of Various Methods of the PAL Spectra Analysis. *Radiat. Phys. Chem.* **2007**, *76* (2), 243. https://doi.org/10.1016/j.radphyschem.2006.03.044.

(15) Hirschmann, E.; Butterling, M.; Hernandez Acosta, U.; Liedke, M. O.; Attallah, A. G.; Petring, M. P.; Görler, M. M.; Krause-Rehberg, R.; Wagner, A. A New System for Real-Time Data Acquisition and Pulse Parameterization for Digital Positron Annihilation Lifetime Spectrometers with High Repetition Rates. *JINST* **2021**, *16* (08), P08001. https://doi.org/10.1088/1748-0221/16/08/P08001.

(16) Olsen, J. V.; Kirkegaard, P.; Pedersen, N. J.; Eldrup, M. PALSfit: A New Program for the Evaluation of Positron Lifetime Spectra. *Phys. status solidi* **2007**, *4* (10), 4004–4006. https://doi.org/10.1002/pssc.200675868.

(17) Pinheiro, M.; Martin, R. L.; Rycroft, C. H.; Haranczyk, M. High Accuracy Geometric Analysis of Crystalline Porous Materials. *CrystEngComm* **2013**, *15* (37), 7531–7538. https://doi.org/10.1039/C3CE41057A.

(18) Zubiaga, A.; Warringham, R.; Mitchell, S.; Gerchow, L.; Cooke, D.; Crivelli, P.; Pérez-Ramírez, J. Pore Topology Effects in Positron Annihilation Spectroscopy of Zeolites. *Chemphyschem* **2017**, *18* (5), 470–479. https://doi.org/10.1002/CPHC.201601258.
